# A Digital Diabetes Prevention Program (Transform) for Adults With Prediabetes: Secondary Analysis

**DOI:** 10.2196/13904

**Published:** 2019-07-26

**Authors:** Meshari F Alwashmi, Gerald Mugford, Waseem Abu-Ashour, Misa Nuccio

**Affiliations:** 1 Memorial University St. John's, NL Canada; 2 Blue Mesa Health New York, NY United States

**Keywords:** mhealth, diabetes, DPP, diabetes prevention program, digital health, diabetes

## Abstract

**Background:**

The prevalence of diabetes is increasing among adults globally. Research has demonstrated that a diabetes prevention program (DPP), which focuses on developing and maintaining health-promoting lifestyle modifications, can prevent or delay the onset of type 2 diabetes among at-risk individuals. The implementation of a digitally adapted DPP has the potential to prevent prediabetes on a national and global scale by using technology and behavior change science.

**Objective:**

This study aimed to investigate the effects of a novel digital therapeutic DPP (Transform) on weight loss, body mass index (BMI), exercise frequency, and work absenteeism.

**Methods:**

This study was a secondary analysis of retrospective data of adults with prediabetes who were enrolled in the Transform DPP from December 2016 to December 2017. The program incorporates interactive mobile computing, remote monitoring, an evidence-based curriculum, behavior tracking tools, health coaching, and online peer support to prevent or delay the onset of type 2 diabetes. The analysis included data that were collected at baseline and after 4 months of the Transform DPP.

**Results:**

The sample (N=273) comprised people with prediabetes who completed 4 months of the Transform program. Participants included 70.3% women, with a mean age of 54.0 (SD 11.2) years. On average, participants decreased their weight by 13.3 lbs (6.5%) and their BMI by 1.9 kg/m^2^. On average, participants increased their exercise frequency by 1.7 days per week, and absenteeism was reduced by almost half a day per month.

**Conclusions:**

These results suggest that the digital therapeutic DPP (Transform) is effective at preventing type 2 diabetes through a significant reduction in body weight and an increase of physical activity. A prospective, controlled clinical study is warranted to validate these findings.

## Introduction

### Background

Diabetes imposes a significant economic burden on society in the form of higher medical costs, lost productivity, premature mortality, and additional costs in the form of disability-adjusted life years [1]. Diabetes is largely attributed to modifiable lifestyle factors such as diet, physical activity, and sleep [2]. There is a need for those at risk of developing type 2 diabetes to engage in health-promoting behaviors to reduce their risk. Specifically, individuals can utilize lifestyle modification programs to reduce weight and increase physical activity [3]. Several published studies suggest that smartphones can deliver effective behavioral interventions among various age groups and for different diseases [4-6]. The purpose of this study was to investigate the effects of a novel digital therapeutic diabetes prevention program (Transform) on weight loss, body mass index (BMI), exercise frequency, and work absenteeism.

In 2017, the International Diabetes Federation (IDF) [7] estimated that, worldwide, approximately 425 million adults, aged 20-79 years, were living with diabetes. By 2045, this number is expected to increase to 629 million. In the United States, it is estimated that more than 30 million adults are living with diabetes [7]. Lin et al [8] projected that the number of adults diagnosed with diabetes in the United States would significantly increase to almost 40 million in 2030 and to more than 60 million in 2060. Based on 79,535 death certificates, diabetes was the seventh leading cause of death in the United States in 2015 [9].

Diabetes imposes a significant economic burden on society. The American Diabetes Association estimated that the total cost of diagnosed diabetes has increased to US $327 billion in 2017 from US $245 billion in 2012 in the United States alone [10]. Indirect costs linked to diabetes in the United States include increased absenteeism (US $3.3 billion) and reduced productivity while at work (US $26.9 billion) [10].

Diabetes is a complex, chronic illness requiring ongoing medical care. It is managed by multifactorial risk-reduction strategies that go beyond glycemic control. Diabetes is caused by several factors including modifiable health behaviors and, in many cases, it is preventable. At-risk individuals can use lifestyle modification programs to reduce weight and increase physical activity [3]. A recent systematic review concluded that lifestyle modifications among individuals with prediabetes reduced the incidence of diabetes development more than standard treatment [11].

#### Mobile Health

The ubiquity of smartphones and tablets has led to the widespread adoption of mobile health (mHealth). The Global Observatory for eHealth of the World Health Organization defines mHealth as “medical and public health practice supported by mobile devices, such as mobile phones, patient monitoring devices, personal digital assistants (PDAs), and other wireless devices” [12]. A mobile health intervention can include the use of home tracking medical devices that are compatible with smartphones and integrate with a health care professional. The significance of mHealth is outlined by its ability to deliver timely care irrespective of the geographical location of the patient and provider. Researchers and clinicians can utilize mHealth to conduct studies aimed at improving the quality and efficiency of health care delivery, enhancing the quality of life, and reducing the overall burden on the health care system. Although mHealth is a nascent industry, it is already showing its potential by significantly enhancing treatment outcomes while mitigating health care costs [13,14].

Several published studies indicate that smartphones can deliver effective interventions [4-6]. Mateo et al [15] conducted a systematic review and meta-analysis to compare the efficacy of mobile phone apps and other approaches that promote weight loss and increase physical activity. They concluded that mobile phone app–based interventions may be useful tools for weight loss [15]. Researchers are proposing mHealth apps for many health conditions such as diabetes, dementia, autism, and dysarthria [16-18]. Research has shown that innovations in health technology demonstrated positive behavior changes among patients with type 2 diabetes [19,20].

#### The Diabetes Prevention Program

Knowler et al [21] conducted a multicenter clinical research study to assess whether moderate lifestyle modifications in the form of dietary changes and increased physical activity could prevent or delay the onset of type 2 diabetes [21]. They concluded that the lifestyle intervention was almost twice as effective at reducing the risk of developing type 2 diabetes as the pharmacological treatment [21]. The results of the study provided a clinical foundation for DPP programs. A study by Hamman et al [22] stated that for every kilogram of weight loss, there was a 16% reduction in type 2 diabetes risk among individuals with prediabetes. Following these findings, the Centers for Disease Control and Prevention (CDC) launched the National Diabetes Prevention Program (NDPP), which includes a lifestyle modifications curriculum that can be delivered setting or online.

The NDPP curriculum consists of an intensive 16-week intervention that uses an evidence-based curriculum, health coaching, social support, and self-monitoring tools to initiate and sustain health behavior changes [23]. The 16-week high-frequency core intervention is followed by 8 months of complimentary maintenance programming to sustain the new lifestyle modifications.

In community-based programs, small groups of at-risk individuals meet on a weekly basis to engage in the curriculum, and work with a trained DPP educator who facilitates the program. The small group setting gives participants the opportunity to express empathy, offer support, and seek support, which are crucial components to behavior change. The group dynamic enables flexibility and tailorability to make the program relevant to each participant. Social support also creates a sense of accountability to others in the group.

The first 8 weeks weeks of the 16-week core curriculum focus on basic tenets of healthy eating and physical activity, while the second half of the core 16-week CDC curriculum focuses on the environmental and social triggers that impact health behavior [24]. Participants are asked to track their food and physical activity daily using paper-based tools and measure their weight weekly at each class. Throughout the 16 weeks, participants aim to lose 5%-7% of their body weight and engage in at least 150 minutes of moderate physical activity each week [24]. The CDC’s NDPP [23] has established a clinical basis for implementing lifestyle interventions in individuals with prediabetes as a reliable and sustainable method of preventing, reversing, or delaying the onset of type 2 diabetes [25].

### Transform—A Digital Diabetes Prevention Program

**Figure 1:**
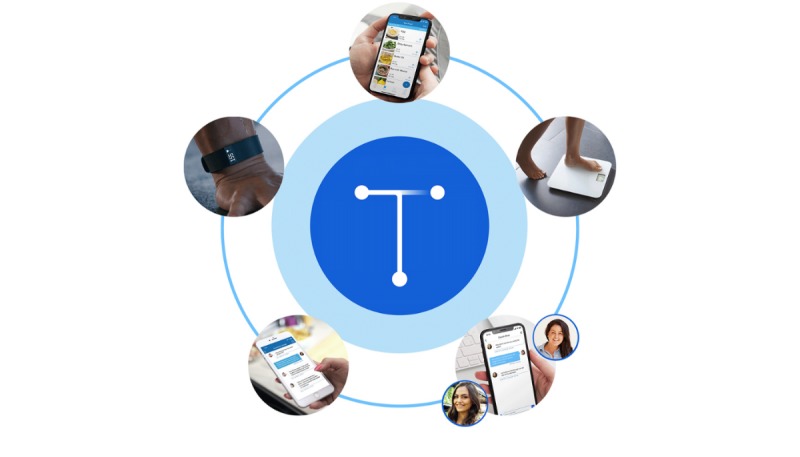
Transform components: a smartphone app with an interactive curriculum, digital tracking and communication tools, a wireless scale, a professional health coach, a private peer community, and an activity tracker.

Blue Mesa Health, a global digital health company based in New York City, adapted the CDC’s DPP to a digital model to enable a scalable, convenient, and flexible delivery of the evidence-based program. Transform integrates interactive mobile computing (eg, smartphone app), wearable tracking devices (eg, activity tracker), remote health monitoring hardware (eg, digital scale), and professional health coaches to effectively address the complex factors that impact behavior. Transform is a 12-month intervention that uses the same two-phase program structure as the CDC’s program: a 16-week high-frequency core intervention followed by 8 months of complimentary maintenance programming to support the new health behaviors. The program components are described in detail below and outlined in Figure 1.

#### Personalized Health Coaching

The program leverages the power of interpersonal connection by matching individuals with DPP-certified health coaches who motivate and guide participants to reach their health goals. Health coaches are trained in diabetes prevention education and use constructs from the Health Belief Model [26] to empower participants to adopt new health behaviors and create long-lasting lifestyle modifications [27]. Through high-frequency interactions and tailored communication, individuals receive empathy and support from their coach as they incorporate new habits into their lives.

Health coaches serve an important moderating and facilitating function by communicating with participants via private messages or calls. They keep participant discussions on track, provide personalized feedback on food logs and physical activity progress, and conduct individualized coaching sessions using specialized techniques such as motivational interviewing.

#### Diabetes Prevention Program Curriculum

The DPP curriculum is presented in a digital format via a smartphone app and includes survey questions, quizzes, and free-response questions. One lesson per week is “unlocked” each Sunday morning in the first 16 weeks, and participants are encouraged to complete the lessons at their convenience within the week. Quiz responses and free responses are shared with the health coach. Lessons are considered complete once a participant completes the quiz.

#### Digital Tracking Tools

In addition to a wearable tracking device and a digital scale, a photo-enabled food diary facilitates tracking of eating behaviors. Participants are asked to track their food intake by taking a picture of each meal, snack, or drink and uploading it to the app. The health coach reviews the tracking weekly and provides feedback. Food tracking enables participants to learn new information and tools to guide their eating patterns and plan meals. Over the course of the program, participants are encouraged to make each meal resemble MyPlate [28].

#### Group Support

To recreate the experience of a group dynamic, which is a core component of the in-person DPP, participants are placed into private chat groups within the smartphone app. An in-app group discussion allows participants to post questions, reply to comments, and share their experience and progress. This component of the Transform program is informed by constructs from Social Cognitive Theory, which empowers participants to improve self-efficacy, increase engagement, and influence behavior [27].

Group discussion is asynchronous, rather than live, to make the intervention more flexible and convenient.

### Objectives

In this study, we aimed to examine the effectiveness of a novel digital DPP (Transform) on weight, BMI, exercise frequency, and work absenteeism in a sample of adults with prediabetes.

## Methods

### Design and Setting

The study is a secondary analysis of data collected via the Transform DPP. Deidentified data that were collected at baseline and 4 months were analyzed. Transform participants were recruited via a marketing channel partner. Participants completed an online eligibility survey that was adapted from the CDC prediabetes screening survey [23]. Eligible participants completed a baseline questionnaire and a 4-month follow-up questionnaire. The questionnaires were adapted from the World Health Organization Health and Work Performance Questionnaire [29] and the National Health and Nutrition Examination Survey [30]. The end of the fourth month was a relevant time point because it marked the end of the first phase (core) of the 12-month DPP.

### Eligibility Criteria

Participants were eligible for the study if they met the following inclusion criteria: score≥9 on the online survey adapted from the CDC prediabetes screening tool [23] or indication of prediabetes diagnosis through a recent blood test (within the last 12 months); BMI≥25 kg/m^2^ or ≥23 kg/m^2^ if self-identified as Asian; age≥18 years; indication of readiness to change based on additional survey questions [31]; and completion of the 4-month follow-up survey and record of body weight during week 16 of the Transform program.

Participants were excluded from the study if they had a BMI<25 kg/m^2^; were <18 years of age; failed to meet at least one of the following criteria: (a) screened as high-risk according to the American Diabetes Association [32] or CDC prediabetes screening tool [23] or (b) self-reported as positive for prediabetes, according to a blood test within the last 12 months; or did not consent for their data to be used for research.

### Measures

Once the participants were accepted into the program, they completed an online baseline health behavior survey to assess their current physical activity, diet, sleep, work performance, and general health status. Participants received a series of onboarding emails and were mailed packages to introduce them to the program. The packages included a wireless scale by BodyTrace, Inc, and a wearable activity tracker by Fitbit, Inc (model: Flex 2). Weight was measured by the wireless scale and height was self-reported; weight and height were both used to calculate BMI. After 4 months, participants completed an online follow-up survey. Four outcomes were assessed: weight, BMI, exercise frequency, and work absenteeism. Work absenteeism was adapted from the World Health Organization Health and Work Performance Questionnaire [29]. Subjects answered the following question: “Think of your work experience over the past 4 weeks (28 days). In the space provided below, type the number of entire workdays missed because of problems with your physical or mental health.” Each measure was calculated as the difference between scores at baseline and 4 months. Participants did not receive an incentive to complete the follow-up survey.

### Ethics

Solutions Institutional Review Board, an independent ethics review company (Little Rock, AR and Yarnell, AZ), reviewed and approved this study.

### Statistical Analysis

Descriptive statistics (mean and SD for continuous variables; frequency and percentage for categorical variables) were used to describe participant demographics. Four paired-samples *t* tests were used to compare the baseline mean BMI, exercise frequency, and work absenteeism with the means at the 4-month follow-up. Analyses were conducted using IBM SPSS Statistics, version 23 (IBM Corp, NY). A *P* value <.05 was considered statistically significant for all tests.

## Results

### Demographics

Of the 1183 individuals who enrolled in the Transform program and completed the baseline survey, 27 participants did not give consent for their data to be used for research purposes. From the participants’ interaction with their coach data, 10.5% (124/1183) of the participants were lost to follow-up. Approximately 23% (n=273) of the participants completed the online follow-up survey at 4 months. This response rate is consistent with other studies using online surveys [33].

The current analysis included all participants that completed the 4-month follow-up survey. A total of 273 subjects were included in the analysis. Of these, 70.3% were female. The mean age of the sample was 54.01 (SD 11.33) years. Participants were primarily white (74%; Table 1).

**Table 1:** Participant demographics (N=273).

Characteristic		Value
Age in years, mean (SD)		54.0 (11.3)
Sex (female), n (%)		192 (70.3)
**Ethnicity, n (%)**		
	White	202 (74)
	Asian	16 (5.9)
	Black	14 (5.1)
	Other	41 (15)

### Outcomes

#### Weight

We observed that the average weight declined by 6.5%. Participants’ mean weight was 205.1 lbs (SD 46.5) at baseline and 191.79 lbs (SD 39.6) at 4 months. There was a statistically significant mean decrease of 13.3 lbs (*P*<.001).

#### Body Mass Index

The number of participants with a recorded BMI at 4 months was 272. There was a significant difference in BMI scores from baseline (mean 32.9 kg/m^2^, SD 6.4 kg/m^2^) to 4 months postintervention (mean 31 kg/m^2^, SD 7.3 kg/m^2^). This was a mean decrease of 1.9 kg/m^2^ (*P*<.001).

#### Exercise Frequency

Data on exercise frequency were available for 202 participants. There was an increase of 1.7 days per week in exercise frequency from baseline (mean 2.4 days, SD 1.8 days) to 4 months (mean 4.1 days, SD 1.7 days; *P*<.001).

#### Work Absenteeism

Work absenteeism data were available for 167 participants. There was a significant difference in the scores from baseline (mean 0.9 days, SD 1.2 days) to 4 months (mean 0.5 days, SD 1.1 days; *P*<.001). On an average, participants decreased their work absenteeism by almost half a day per month.

## Discussion

### Principal Findings

This secondary analysis demonstrates that adults with prediabetes who used a novel and individualized digital diabetes prevention program had significant reductions in BMI, weight, and work absenteeism. They also demonstrated a significant increase in exercise frequency at the end of the 4-month study period. This strongly suggests that the Transform digital DPP is an effective tool to drive positive changes in lifestyle behaviors associated with prevention of diabetes.

The Finnish National Diabetes Prevention Program implemented its DPP program in-person through clinics [34]. They were able to achieve an average weight loss of 1.2% among 919 participants, which is less than the weight loss recorded in our study. Although this study supports the findings of other digital interventions targeting diabetic patients [35,36], the current study utilizes a comprehensive digital DPP targeting adults with prediabetes.

Among participants with prediabetes using the Transform DPP, the mean weight declined by 6.5%. This amount of weight loss can be interpreted as a clinically significant reduction in diabetes risk. According to Knowler et al, 5% to 7% of body weight loss reduces the risk of developing type 2 diabetes by 58% in adults with prediabetes and by 71% for people over 60 years old [21]. Additionally, Hamman et al [22] stated that for every kilogram of weight loss, there was a 16% reduction in diabetes risk. Colditz et al [37] noted that loss of 1 kg of weight was associated with a 10% reduction in diabetes risk.

### Limitations and Strengths

This study was observational and not experimental. It lacks a control group, which may help in addressing possible confounders. This study does not investigate the individual contribution of each program component on the outcome variables. Another limitation was the reliance on self-reports of exercise frequency and work absenteeism. Bias in self-reported physical activity can be avoided in the future by using data from an activity tracker.

The main strength of this study is that it is a secondary analysis of real-world implementation of the intervention. Participants did not receive an incentive to complete the follow-up survey. A benefit of real-world evidence is the generalizability of the outcomes.

### Future Research

Data on outcome sustainability after 4 months were not collected in this analysis. Future studies should examine 12-24 months of follow-up data to address the sustainability of behavior change following the intervention. Future research should include an experimental study to assess possible confounding variables including ethnic origin, education, socioeconomic factors, gender, and other factors related to the outcomes such as reduction in hemoglobin A1c levels. Future research would benefit from the inclusion of an economic analysis of the impact of the Transform DPP and should consider both the direct and indirect costs.

### Conclusions

Transform, a digital DPP adapted from the National Diabetes Prevention Program, was developed to prevent or delay the onset of type 2 diabetes in adults with prediabetes. Transform incorporates interactive mobile computing, remote monitoring, health coaching, evidence-based curriculum, behavior tracking tools, health coaching, and online peer support to prevent or delay the onset of type 2 diabetes. We observed that the Transform DPP significantly reduces BMI, body weight, and work absenteeism and increases exercise frequency. The study findings highlight the effectiveness of the Transform program.

## Abbreviations

BMI: body mass index
CDC: Centers of Disease Control and Prevention
DPP: diabetes prevention program
IDF: International Diabetes Federation
mHealth: mobile health
NDPP: National Diabetes Prevention Program
PDA: personal digital assistant
